# Parkin Pleiotropy: Extremely Atypical Phenotypes in Patients With Compound Heterozygous Mutations

**DOI:** 10.5334/tohm.55

**Published:** 2020-08-13

**Authors:** Patricio Millar Vernetti, Malco Rossi, Marcelo Merello

**Affiliations:** 1 Movement Disorders department, Fleni, Buenos Aires, AR; 2 National Scientific and Technological Research Council (CONICET), Buenos Aires, AR; 3 Pontificia Universidad Católica Argentina (UCA), Buenos Aires, AR

**Keywords:** Movement disorders, Parkin, Whole Exome Sequencing, Atypical parkinsonisms

## Abstract

**Background:**

Parkin mutations are suspected in early-onset Parkinson’s disease with early motor complications, and in pedigrees showing an autosomal recessive pattern. Some compound heterozygous mutations can present with various uncommon phenotypes.

**Case Report:**

Two siblings with the same mutations, one with atypical postural and action tremor, and the other with an axonal motor autonomic neuropathy. A woman with a 45-year history of slowly progressive parkinsonism with no motor complications.

**Discussion:**

Due to the variability of phenotypes of Parkin mutations, testing should also be warranted in patients with atypical tremor syndromes or axonal polyneuropathy when more common causes have been ruled out.

**Highlights:**

We report three patients with extremely atypical parkin mutation phenotypes: an atypical tremor syndrome, an axonal motor autonomic neuropathy, and a remarkably slowly progressive parkinsonism. This shows that parkin mutations may present with a highly variable phenotype, and should be considered in patients with such manifestations.

## Introduction

Homozygous mutations in the Parkin gene have been originally recognized to cause Parkinson’s disease (PD; PARK-Parkin) in patients with early disease onset and a phenotype closely resembling idiopathic PD, but with rapidly developing levodopa-induced dyskinesia and motor fluctuations, as well as a striking response to anticholinergic medication [1]. As techniques for genetic diagnosis became more available and refined, the genotypic spectrum of PARK-Parkin has been expanded to compound heterozygous cases encompassing patients with different phenotypic presentations [2]. Patients may initially present with different types of isolated dystonia such as lower limb kinesiogenic dystonia [3], cervical dystonia or task-specific dystonia (which are more frequent in younger patients) and may have a higher frequency of psychiatric disturbances [4,5].

Here we present three patients with compound heterozygous Parkin mutations, diagnosed by whole exome sequencing (WES) and confirmed by Sanger sequencing; two of them siblings with the same mutations, and all of them presenting with different and highly unusual phenotypes.

## Case description

### Case 1

A 23-year-old boy, brother of Case 2, with no history of toxic or drug exposure, started at age 14 with action tremor in the right arm. Three years later, at the age of 17, he consulted us for a worsening of the tremor and progression to all four limbs. He denied REM sleep behaviour disorder, hyposmia, depression or constipation. On physical examination the patient exhibited postural and action tremor in both arms, with an asymmetrical rest component, of greater amplitude in the right side; and rest tremor in both legs, of greater amplitude in the left side (Video 1). He also had minimal dystonic posturing in his right arm and foot. As the patient underwent complementary studies, both an acute levodopa challenge and one-month 1000 mg/day trial were performed to look for a possible therapeutic agent and narrow down differential diagnosis, but tremor was neither responsive to this, nor to alcohol nor propranolol. 18F-DOPA-PET scan showed a normal striatal uptake. Olfactory function assessment proved normal. Other complementary studies were normal, including brain MRI, complete blood count, liver function tests, homocysteine, B12, folate, VDRL, thyroid screening, serum copper, 24-hour cupruria, ceruloplasmin, EKG and nerve conduction studies. Fragile-X pre-mutations and Charcot Marie Tooth type 1A mutations were also investigated due to his sibling’s phenotype and proved normal. Whole exome sequencing (WES) detected a heterozygous pathogenic variant in exon 7 of the Parkin gene at c.823C>T (p.Arg275Trp) and an unreported heterozygous variant in the acceptor splice site of intron 4 at c.535-2A>C, categorized as likely pathogenic by in silico parameters due to a very likely aberrant effect on splicing. The patient received trihexyphenidyl 5 mg TID with almost complete tremor resolution and sustained response for the last five years. He has neither developed additional parkinsonian features nor autonomic dysfunction.

**Video 1:** **Case 1.** Case 1, age 23, shows rest and postural tremor in four limbs, predominantly right upper and left lower limbs. The patient shows no signs of bradykinesia. Dystonic posturing presents as flexion of the right arm and plantar flexion of the right foot.

### Case 2

**Figure 1:**
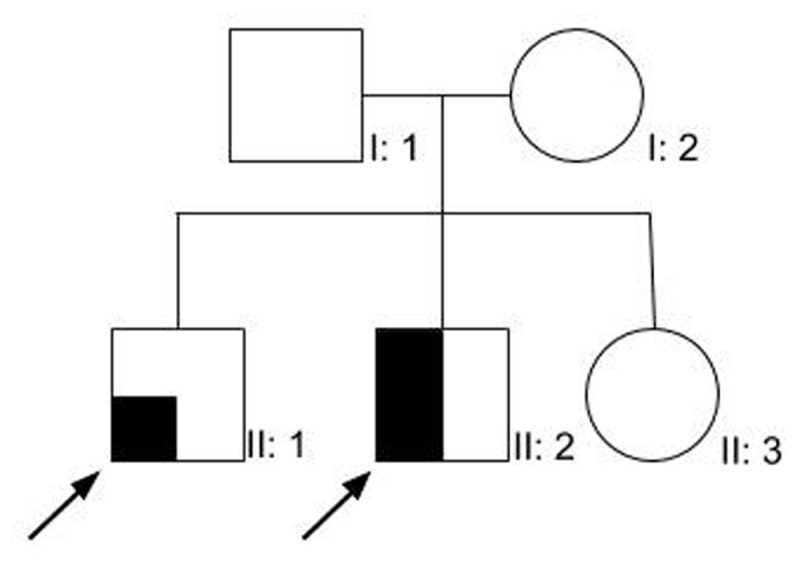
Family tree for cases 1 and 2.

The younger brother of patient 1 (Figure 1), complained of lower limb weakness since the age of 12. Physical examination revealed 4/5 muscular weakness both proximal and distal in four limbs, with no major functional compromise, and without sensitive alterations. At the same age, he developed repetitive syncope, and also presented chronic constipation. Neurological examination revealed bilateral mild intention and postural hand tremor. Nerve conduction studies revealed an axonal motor polyneuropathy, predominantly in the lower limbs, and tilt test evidenced orthostatic hypotension. He had no other findings on complementary studies such as case 1, including 18F-DOPA-PET scan. WES found the same mutations than his elder brother depicted in Case 1. Tremor exhibits a slight progression and no additional parkinsonian features have appeared in the last five years, nor the need for symptomatic treatment. At the age of 15, his muscular strength remains stable and by means of a rehabilitation program, he is able to follow a normal lifestyle with no medication.

### Case 3

A 65-year old woman began at the age of 20 with rest tremor in the right hand and subtle rigidity and bradykinesia in the right arm, which were non-progressive for more than ten years. Her family history revealed that her father suffered PD since the age of 60. When first seen by us at the age of 39, she presented a severe bilateral and symmetric rest, postural, and intention jerky tremor also involving the head but not her chin. She presented slight rigidity and bradykinesia. Brain MRI was normal, as well as 24-hour cupruria. Olfactory testing was normal, and dopaminergic imaging with 99mTc-TRODAT-1 revealed a mild bilateral reduction uptake, with a posterior to anterior gradient, predominantly on the right putamen. Tremor was unresponsive to neither 250 mg acute challenge with levodopa nor to sustained 1000 mg/day dose for more than one month. Trihexyphenidyl 5 mg TID resulted in a marked reduction of tremor. After ten years of use, she developed cognitive complaints that completely reverted with trihexyphenidyl withdrawal. The patient exhibited a remarkably mild motor compromise, with mildly more prominent bradykinesia in the left side of her body, consistent with the 99mTc-TRODAT-1 findings (Video 2). The patient has remained on monotherapy with dopamine agonists for the last 20 years, currently with 3 mg of pramipexole. At the age of 65, with 45 years of disease duration, she has neither developed drug-induced dyskinesias nor wearing-off phenomena. Her condition has slightly progressed due to the development of freezing of gait. Sporadic attempts to reintroduce levodopa have showed no response in tremor or in the freezing of gait. WES detected a known heterozygous pathogenic variant in exon 2 of the Parkin gene at p.(Asn52Metfs*29) and a known heterozygous pathogenic variant in exon 7 at p.(Arg275Trp).

**Video 2:** **Case 3.** Case 3, age 65, shows a jerky resting, postural and intention tremor, of greater amplitude in the right side; and mild bradykinesia, predominantly in the right side. Video was taken after a 24 hour withdrawal of dopaminergic agonists.

## Discussion

Although the phenotypic variation of compound heterozygote Parkin mutation has been described [2] none of the previously reported cases have displayed such unusual features as these three cases. Two cases shared the same compound-heterozygous mutations including a previously unreported variant in intron 4 at c.535-2A>C. The latter of which was not present in their asymptomatic sister (Figure 1) who is a carrier of the exon 7 mutation, indicating that the detected variants are in compound heterozygous state. Even though the pathogenicity of the said variant was classified as likely pathogenic due to a very likely aberrant effect on splicing by *in silico* parameters, there is currently no data available on these mutations’ frequencies in controls in South America, and the patients’ RNA was not evaluated to demonstrate an effect on mRNA splicing. Nevertheless, their asymptomatic sister is a carrier of the exon 7 mutation, but not the one in intron 4, which supports the pathogenicity of said unreported variant. Even though the patients had normal 18-F-DOPA scans, this does not preclude the diagnosis of genetic neurodegenerative disorders [6]. Some spinocerebellar ataxias may present with parkinsonism and neuropathy, and although we did not test for repeat expansion disorders, these features were only present individually in each patient, and not in combination. Furthermore, neither of them had pyramidal, cerebellar or oculomotor features, and even though the patients had an early onset of the disease (which could indicate a higher number of repeats in a disorder which usually has a late onset), they did not have any other family members affected. And lastly, they had a mutation in a gene known to present as either parkinsonism or neuropathy on their own. Parkin mutations are known to be associated with peripheral axonal neuropathies, mostly as an incidental finding on EMG examination, although less commonly as part of the clinical picture, and even more infrequently as the initial symptom, later progressing with parkinsonian features as well [7,8,9,10], however, not described as a major manifestation of the disease (Case 2). In Case 2, the patient exhibited predominant symptoms associated with an axonal motor neuropathy with autonomic involvement as his major clinical manifestation, which was not present in his eldest brother with the same mutation. Likewise, although adduction-abduction tremor in the lower limbs [8] has been described, our patient in case 1 is the first report of such a tremor occurring in the upper extremities, as well as case 3 who presented with an unreported “jerky” tremor. Even though case 1 had a prominent tremor syndrome, a levodopa trial was attempted in order to assess for a potential therapeutic agent and narrow down the differential diagnosis as part of an initial evaluation as the patients undergo other complementary studies and await their results.

In addition, none of the cases have fulfilled clinical criteria for PD despite several years of disease duration, nor have they developed psychiatric complications, although in the case of the siblings, these could be expected to develop over the years, and we will continue to periodically monitor their evolution. Another major finding of the current cases is the lack of response to sustained doses of L-dopa but a striking and sustained response to anticholinergics, consistent with their tremor-dominant phenotype, which appear to improve symptoms of PD through a central anticholinergic effect exerted in the striatum [11]. This might suggest post-synaptic affectation of Parkin mutation [12].

Discordant phenotypes in siblings sharing the same genotype has been reported in patients with similar motor features, as the case of two sisters with hemiparkinsonism and hemidystonia, one of which presented cognitive dysfunction [13], or two siblings who presented with isolated lower limb dystonia, but only one developing parkinsonian features on follow up [14]. However, the striking characteristics of cases 1 and 2, is that they share almost no clinical feature. While the “classical parkin” phenotype is that of a mild and slowly progressive parkinsonism, patients often develop motor fluctuations and dyskinesia very early during the course of the disease, ranging from as soon as 3 days to up to 21 years [5]. One of the patients described here spent her 25 first years after disease onset without treatment and has since been treated for over 20 years with DAs without the need of adding levodopa, or having developed motor complications.

Even though some phenotypic characteristics such as an earlier onset and more severe course can be attributed to certain genotypic changes such as missense mutations or the presence of multiple mutations [4], the variability of phenotypes of Parkin mutations even within families with the same mutation reinforces the role of epigenetic factors in disease expression and is a clear example of pleiotropy in genes inducing parkinsonism. Moreover, the presence of only one mutation in the so-far asymptomatic sister of cases 1 & 2, supports the pathogenic role of cumulative mutations in compound heterozygous patients. This underscores the importance of reporting atypical cases to bring attention to help identify future cases and eventually help determine their pathogenicity and possible clinical and genetic implications. Thus, Parkin mutation analysis should be warranted in patients with early-onset atypical tremor syndromes or axonal polyneuropathy when other more common causes have been ruled out or in a setting of a family history of unusual tremor or parkinsonism.
